# Topological insulator metamaterials with tunable negative refractive index in the optical region

**DOI:** 10.1186/1556-276X-8-526

**Published:** 2013-12-13

**Authors:** Tun Cao, Shuai Wang

**Affiliations:** 1 Department of Biomedical Engineering, Dalian University of Technology, Dalian 116024, People's Republic of China

**Keywords:** Metamaterials, Metal-insulator-metal structures, Surface plasmons, 81.05.Xj, 73.40.Rw, 73.20.Mf

## Abstract

A blueshift tunable metamaterial (MM) exhibiting a double-negative refractive index based on a topological insulator (bismuth selenide, Bi_2_Se_3_) has been demonstrated in the near-infrared (NIR) spectral region. The potential of Bi_2_Se_3_ as a dielectric interlayer of the multilayer MM is explored. The optical response of elliptical nanohole arrays penetrating through Au/Bi_2_Se_3_/Au films is numerically investigated using the finite difference time domain (FDTD) method. The blueshift tuning range of the MM is as high as 370 nm (from 2,140 to 1,770 nm) after switching the Bi_2_Se_3_ between its trigonal and orthorhombic states.

## Background

Metamaterials (MMs) are artificially engineered composites that attract considerable interests due to their exceptional electromagnetic properties, which are not typically found in nature, such as negative refractive index and cloaking [1-4]. These MMs with various subwavelength resonant elements have offered magnetic and/or electric resonant responses to incident electromagnetic radiation, scalable from the microwave frequencies up to the terahertz and optical ones [5-7]. Particularly, nanohole resonators embedded in metal-dielectric-metal (MDM) multilayers are frequently used as building blocks of negative-refractive-index MMs [8-11], owing to the coupling between surface plasmons counterpropagating on the two closely spaced interfaces which results in a closed loop of the electric currents. This gives rise to magnetic dipolar resonances between the two coupled metal layers, while the continuous metallic strip parts provide the electric resonance moments [12,13]. All these features make the nanohole array perforating through MDM films become a strong candidate for developing three-dimensional negative-index MMs [14,15].

One of the obstacles in this progress is the resonance responses of MMs to the impinge light which are usually fixed once the dimension of the structure is determined, thus making the MMs possess a limited bandwidth. However, for many applications (switching, modulation, filtering, etc.), it would be highly desirable to tune the MM resonances over a wide bandwidth. To this end, tunable photonic MMs, the spectral range of which can be controlled by changing the dielectric environment of the resonator with liquid crystals (LCs) [16-18]; phase transition materials [19,20]; and optical pumping [21,22] have been discussed recently. However, the challenge is to develop tunable MDM-MMs in the near-infrared (NIR) regime. It is due to the fact that frequency tunability of the MDM-MM mainly requires for the interlayer dielectric material to possess a tunable effective dielectric constant in the NIR region, hence limiting the choice of the active materials. Here, we take a different approach to actively tune the resonant frequency of the MDM-MMs in the NIR regions by using bismuth selenide (Bi_2_Se_3_) as the dielectric layer.

Recently, a rising Dirac material - topological insulators (TIs) - had been intensively researched in condensed matter physics [23,24]. In analogy to the optoelectronic applications of graphene, a thin layer of TIs has been theoretically predicted to be a promising material for broadband and high-performance optoelectronic devices such as photodetectors, terahertz lasers, waveguides, and transparent electrodes [25]. Among these TIs, Bi_2_Se_3_ is a particularly interesting compound due to its relatively large bulk band gap and a simple surface state consisting of a single Dirac cone-like structure [26,27]. Study of the dielectric function reveals that the optical dielectric constant of Bi_2_Se_3_ can be very different for the trigonal and orthorhombic phases in the NIR regime [28]. Bi_2_Se_3_ exhibits a number of means through which their dielectric properties can be altered [28-33]. Herein, structural phase transition between trigonal and orthorhombic states, which is achieved by a high pressure and temperature, is proposed and studied as a means to change the intrinsic effective dielectric properties of the MDM-MMs [28].

Here, we numerically demonstrate a blueshift tunable nanometer-scale MM consisting of an elliptical nanohole array (ENA) embedded in the MDM multilayers where the dielectric core layer is a Bi_2_Se_3_ composite. Under a high pressure of 2 to 4.3 Pa at 500°C, Bi_2_Se_3_ occurring in trigonal phase undergoes a transition to orthorhombic phase and features a large change in the values of the effective dielectric constant [28]. Accordingly, a massive blueshift of the resonant response (from 2,140 to 1,770 nm) of a Bi_2_Se_3_-based MDM-ENA is achieved in the NIR region. Our proposed blueshift tunable negative-index MM provides greater flexibility in the practical application and has a potential of enabling efficient switches and modulators in the NIR region.

## Methods

**Figure 1:**
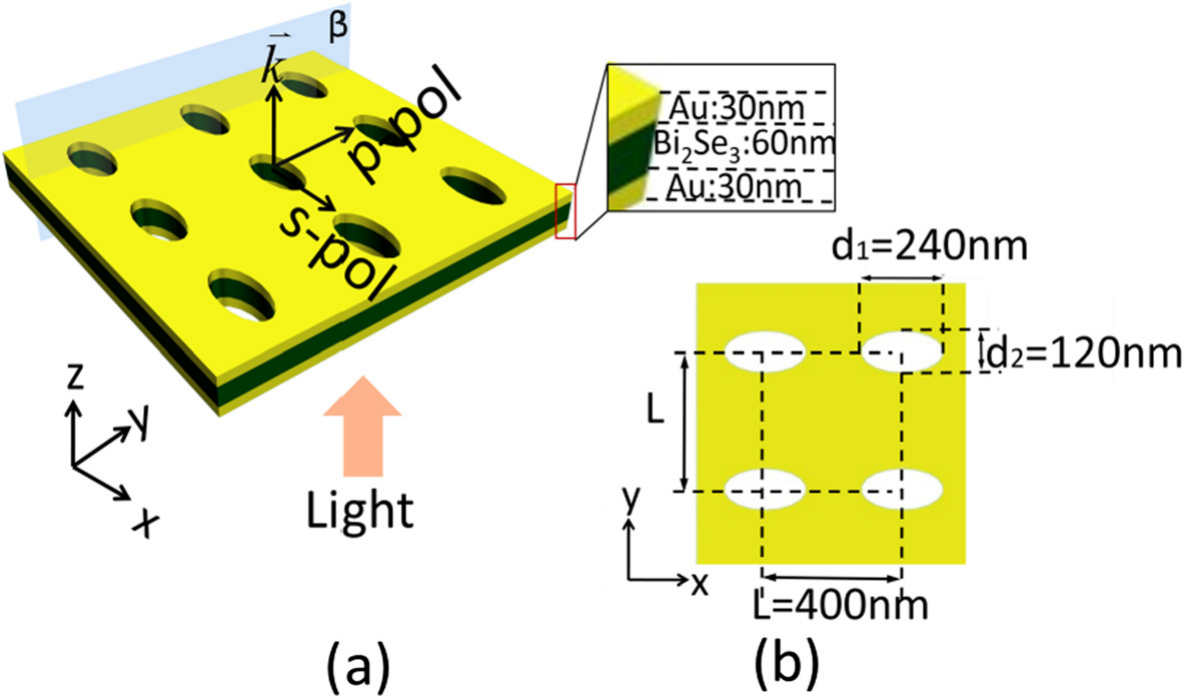
**Schematic of the proposed structure. (a)** Schematic of the MDM structure consisting of a 60-nm-thick Bi_2_Se_3_ dielectric layer between two 30-nm-thick Au films perforated with a square array of elliptical holes suspended in air. The lattice constant is *L* = 400 nm, and hole diameters are *d*_1_ = 240 nm and *d*_2_ = 120 nm. **(b)** Illustration of the square lattice of ENA.

The proposed MDM-ENA suspended in a vacuum is shown in Figure 1, with the coordinate axes and the polarization configuration of the normally incident light. The structure consists of trilayers of Au/Bi_2_Se_3_/Au. The thickness of each Au layer is 30 nm, and the thickness of the Bi_2_Se_3_ layer is 60 nm. The metamaterial parameters are optimized for the maximum sensitivity of the resonance to a change in the refractive index of the Bi_2_Se_3_ core dielectric layer in the NIR spectral range. The element resonator is shown in Figure 1b, where the pitch of the elliptical holes is *L* = 400 nm, the diameters of the elliptical holes are *d*_1_ = 240 nm and *d*_2_ = 120 nm, and *β* is a cross-sectional plane of the structure. The *z*-axis is normal to the structure surface, and the *x*-*y* plane is parallel to the structure surface. This simulated structure is periodically extended along the *x* and *y* axes. The tunable optical properties of the structure are calculated using 3D EM Explorer Studio [34], a commercial finite difference time domain (FDTD) code. In the simulation, a simple Drude-type model for Au permittivity was used, which is a good approximation to experimental values in the NIR region. The dielectric properties of Au as given by Johnson and Christy are used [35]. A plane wave source is simulated at normal incidence to the structure. The computational domain (400 nm × 400 nm × 1,000 nm) has a perfectly matched layer (PML), absorbing boundaries in the *z* direction and periodic boundaries in the *x*-*y* plane [36]. A uniform FDTD mesh size is adopted. The mesh size is the same along all Cartesian axes: *∆x* = *∆y* = *∆z* = 2 nm, which is sufficient to minimize the numerical errors arising from the FDTD method.

**Figure 2:**
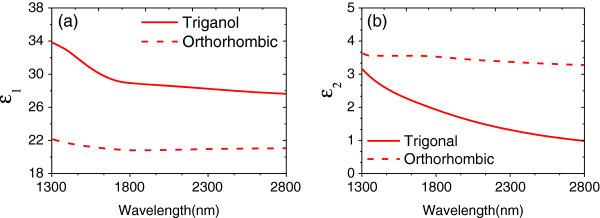
**Dielectric constant of the Bi**_**2**_**Se**_**3**_**. (a)** Real part of dielectric function *ϵ*_1_(*ω*) for trigonal and orthorhombic phases of Bi_2_Se_3_. **(b)** Imaginary part of dielectric function *ϵ*_2_(*ω*) for trigonal and orthorhombic phases of Bi_2_Se_3_.

The topological insulator material Bi_2_Se_3_ was selected due to its significantly different optical properties between the trigonal and orthorhombic phases. The real (*ϵ*_1_) and imaginary (*ϵ*_2_) parts of the dielectric function for the different structural phases of Bi_2_Se_3_ were obtained from the published data in [28]; the NIR spectral region is shown in Figure 2. A large change in the dielectric function across the NIR is obtained after switching Bi_2_Se_3_ from trigonal to its orthorhombic phase.

After the complex coefficients of transmission $t=T_{a}e^{i\varphi_{a}}$ and reflection $r=R_{a}e^{i\varphi_{\mathrm{ra}}}$ are obtained by the 3D EM Explorer Studio, in which *T*_*a*_ is the amplitude and *φ*_*a*_ is the phase of the transmission coefficient, and *R*_*a*_ is the amplitude and *φ*_*ra*_ is the phase of the reflection coefficient, the effective optical parameters can be extracted using the Fresnel formula [37].

For an equivalent isotropic homogenous slab of thickness *h* surrounded by semi-infinite media with refractive index *n*_1_ and *n*_3_ under normal incidence, we have

(1) $$\eta =\pm \sqrt{\frac{{\left(1+r\right)}^{2}-t^{2}}{n_{1}^{2}{\left(1-r\right)}^{2}-n_{3}^{2}t^{2}}}$$

(2) $$n_{\text{eff}}=\pm \frac{1}{\text{kh}}\text{arccos}\left[\frac{1}{t}\frac{n_{1}\left(1-r^{2}\right)+n_{3}t^{2}}{n_{1}+n_{3}+r\left(n_{3}-n_{1}\right)}\right]+\frac{2\mathrm{\pi m}}{\text{kh}}.$$

The so-called material parameters *ϵ*_eff_ and *μ*_eff_ of a single layer of a double-fishnet negative-index metamaterial are extracted using the well-known Nicholson-Ross-Weir (NRW) method [38-40]. Therefore, once *n*_eff_ and *η* are evaluated, the effective permittivity and permeability are calculated using

(3) $$\epsilon_{\text{eff}}=n_{\text{eff}}/\eta ,\;\mu_{\text{eff}}=n_{\text{eff}}\eta ,$$

where *n*_eff_ is the effective refractive index, *η* is the impedance, *h* is the thickness of the structure, *k* = *ω*/*c*, *c* is the speed of light, *m* is an arbitrary integer, and *n*_1_ = *n*_3_ = 1 since the structure is suspended in a vacuum. The signs of *n*_eff_ and *η* and the value of *m* are resolved by the passivity of the metamaterial that requires the signs of the real part of impedance *η* and imaginary part of effective index *n*_eff_ to be positive, i.e., Real(*η*) > 0, Imag(*n*_eff_) > 0 which is consistent with the study described in [39,40]. We then apply this extraction approach to determine the change in the optical response of the structure when the phase of Bi_2_Se_3_ is switched between its trigonal and orthorhombic states.

## Results and discussion

**Figure 3:**
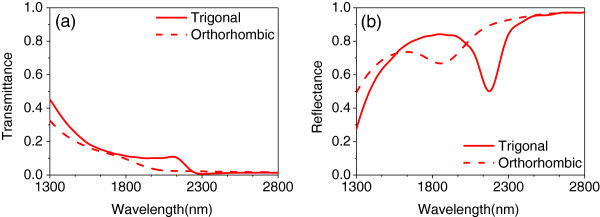
**Transmittance and reflectance.** 3D FDTD simulation of **(a)** spectrum of transmittance and **(b)** spectrum of reflectance, for the different phases of the Bi_2_Se_3_ dielectric layer, where the light source is *p* polarization at normal incidence angle.

The ENA has a lower transmittance for *s*-polarized light due to the electric field's orientation with respect to the metallic stripe width [12]; hence, the polarization of the incident wave was set to be *p*-polarized. As shown in Figure 1a, *s* polarization means that the incident electric field vector is parallel to the long axis of the ENA, and the incident electric field vector perpendicular to the long axis of the ENA is then denoted by *p* polarization. We first investigate the transmittance *T* = |*t*|^2^ and reflectance *R* = |*r*|^2^ of the structure for *p* polarization in Figure 3. Structures with a different dielectric constant of Bi_2_Se_3_ (shown in Figure 2) were modeled to investigate the effect of the phase change of Bi_2_Se_3_ on the position and amplitude of the spectrums. It can be seen that the resonance wavelength blueshifts from 2,140 to 1,770 nm when the structural phase of Bi_2_Se_3_ switches from trigonal to orthorhombic. The structure is impedance-matched, hence possessing a low reflectance corresponding to the dips in reflectance of Figure 3b for different forms of Bi_2_Se_3_.

**Figure 4:**
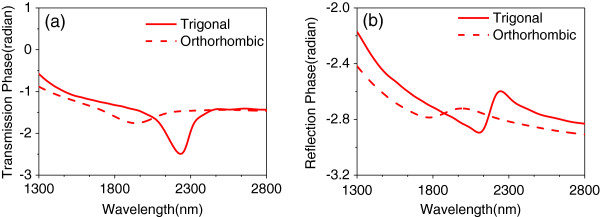
**Transmission and reflection phase.** 3D FDTD simulation of **(a)** phase of transmission and **(b)** phase of reflection, for the different phases of the Bi_2_Se_3_ dielectric layer, where the light source is *p* polarization at normal incidence angle.

In Figure 4, the transmission (*t*) and reflection(*r*) phases are demonstrated. The transmission phase exhibits a dip around the resonance, indicating that the light is advanced in phase at the resonance, characteristic of a left-handed material [41]. Importantly, changing the structural phase of the Bi_2_Se_3_ offers transmission and reflection phase tunability which implies tunable effective constitutive parameters in the structure.

**Figure 5:**
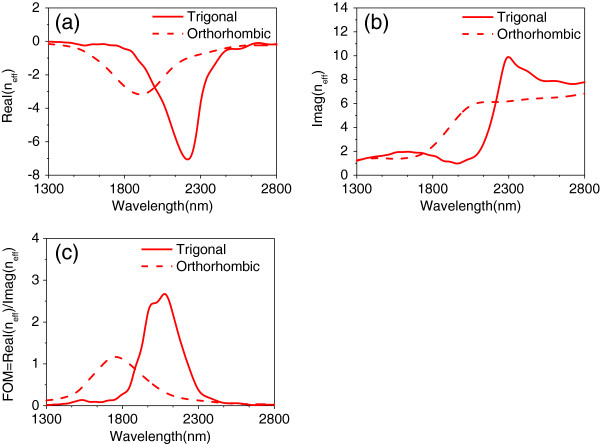
**Effective index and figure of merit.** 3D FDTD simulation of **(a)** real part of *n*_eff_, **(b)** imaginary part of *n*_eff_, and **(c)** figure of merit for the different phases of the Bi_2_Se_3_ dielectric layer, where the light source is *p* polarization at normal incidence angle.

Taking into account the subwavelength thickness of the structure, the extracted *n*_eff_ can be retrieved from the transmission and reflection coefficients shown in Figure 5. For the MM with the trigonal Bi_2_Se_3_ dielectric layer, the negative-index band extends from 1,880 to 2,420 nm with a minimum value of the real part of the refractive index Real(*n*_eff_) = -7. Regarding losses, the figure of merit (FOM) defined as $\text{FOM}=\frac{\text{Real}\left(n_{\text{eff}}\right)}{\text{Imag}\left(n_{\text{eff}}\right)}$ is taken to show the overall performance of the MM, where Imag(*n*_eff_) is the imaginary part of the refractive index. As shown in Figure 5c, the FOM for the trigonal phase is 2.7 at the operating wavelength of 2,080 nm. The negative-index band of the orthorhombic Bi_2_Se_3_-based MM extends from 1,600 to 2,214 nm having a minimum value of Real(*n*_eff_) = -3.2. The FOM is 1.2 at the resonant wavelength of 1,756 nm. Furthermore, the bandwidth of Real(*n*_eff_) becomes wider for the orthorhombic Bi_2_Se_3_ film in Figure 5a due to increased damping of the plasmon resonance but at the cost of an accompanied lower value of FOM in Figure 5c.

**Figure 6:**
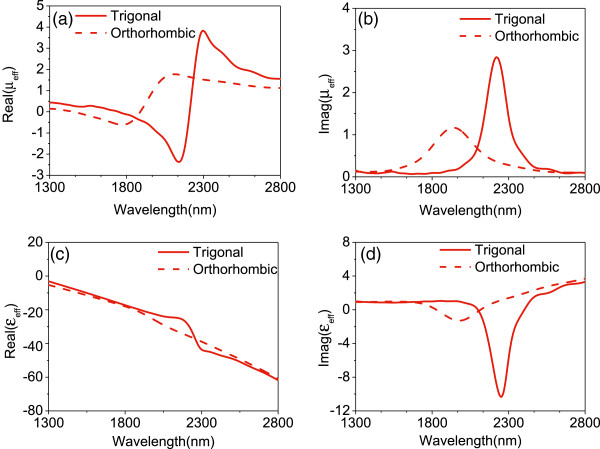
**Permittivity and permeability.** 3D FDTD simulation of **(a)** the real part of *μ*_eff_, **(b)** the imaginary part of *μ*_eff_, **(c)** the real part of *ϵ*_eff_, and **(d)** the imaginary part of *ϵ*_eff_ for the different phases of the Bi_2_Se_3_ dielectric layer, where the light source is *p* polarization at normal incidence angle.

The refractive index is expressed in terms of the real and imaginary parts of the permeability *μ*_eff_ and permittivity *ϵ*_eff_. However, the sign of the real part of the permeability *μ*_eff_: Real(*μ*_eff_) determines the relative magnitudes of the imaginary and real parts of the refractive index [41]. To achieve a negative index with a small loss, a negative Real(*μ*_eff_) is required. Therefore, we have simulated *μ*_eff_ and *ϵ*_eff_ for the structure as shown in Figure 6. For the trigonal and orthorhombic phases of Bi_2_Se_3_, Real(*μ*_eff_) has a Fano-type line shape and Im(*μ*_eff_) has a Lorentzian line shape in the region of the negative index. Moreover, a double-negative MM can be achieved when Real(*μ*_eff_) and Real(*ϵ*_eff_) simultaneously reach negative values over a wide frequency range and thus a reduced loss. The maximum negative Real(*μ*_eff_) decreases with the phase transition from the trigonal to orthorhombic, hence resulting in the smaller value of the maximum negative Real(*n*_eff_) at the orthorhombic phase.

**Figure 7:**
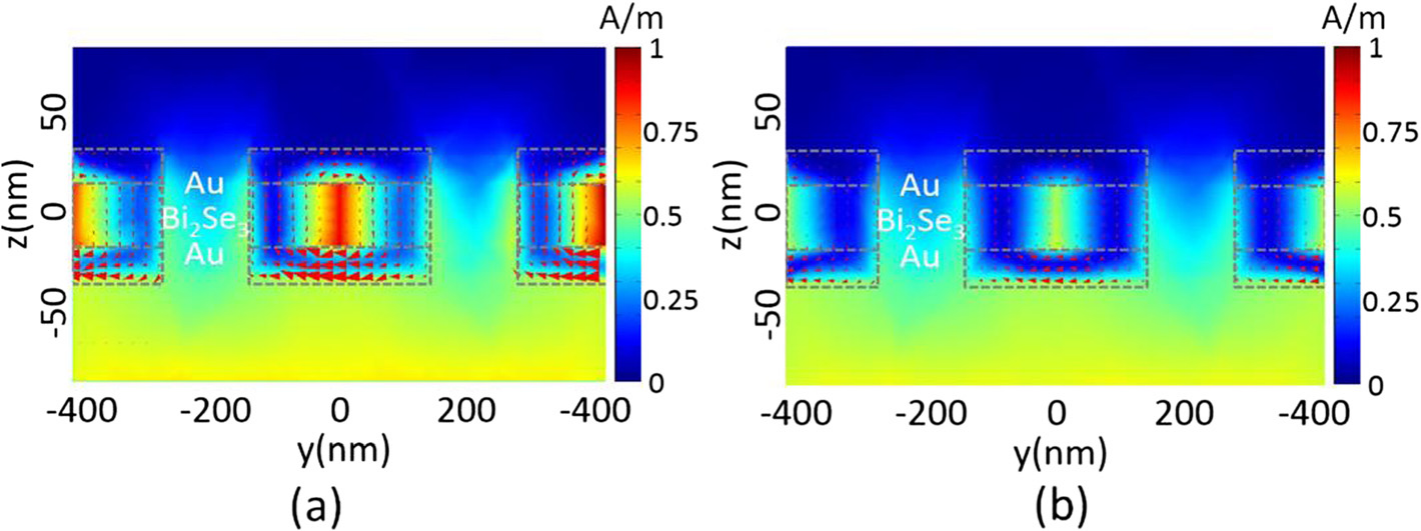
**Magnetic field intensity and displacement current.** A map of the normalized total magnetic field intensity distribution (color bar) and displacement current (*J*_D_) (arrows) along the plane *β*: **(a)** at a 2,140-nm resonance wavelength for trigonal Bi_2_Se_3_ and **(b)** at a 1,770-nm resonance wavelength for orthorhombic Bi_2_Se_3_, where the light source is *p* polarization at normal incidence angle.

This magnetic negative response can be explained looking at the current and field distribution at the resonance wavelengths. Figure 7 shows the current and total magnetic field intensity $H=\sqrt{{\left|H_{x}\right|}^{2}+{\left|H_{y}\right|}^{2}+{\left|H_{z}\right|}^{2}}$ for the magnetic resonant wavelengths of 2,140 and 1,770 nm at the *β* plane shown in Figure 1. In the field maps of Figure 7, the arrows show the currents, whereas the color shows the intensity of the magnetic field. It clearly shows that the antiparallel currents are excited at opposite internal metallic interfaces, closed by an electric displacement current *J*_D_. Therefore, these virtual current loops between two Au layers on the *β* plane give rise to magnetic resonant responses of negative Re(*μ*_eff_) that interact strongly with the incident magnetic field at which the total magnetic field intensity *H* is strongly localized in the Bi_2_Se_3_ dielectric layer between the top and bottom Au layers [42].

Specifically, *H* for the orthorhombic phase shown in Figure 7b is weaker than the trigonal phase shown in Figure 7a. It depicts that the MM based on orthorhombic phase has a smaller magnetic dipolar moment than the trigonal phase and thus smaller FOM.

**Figure 8:**
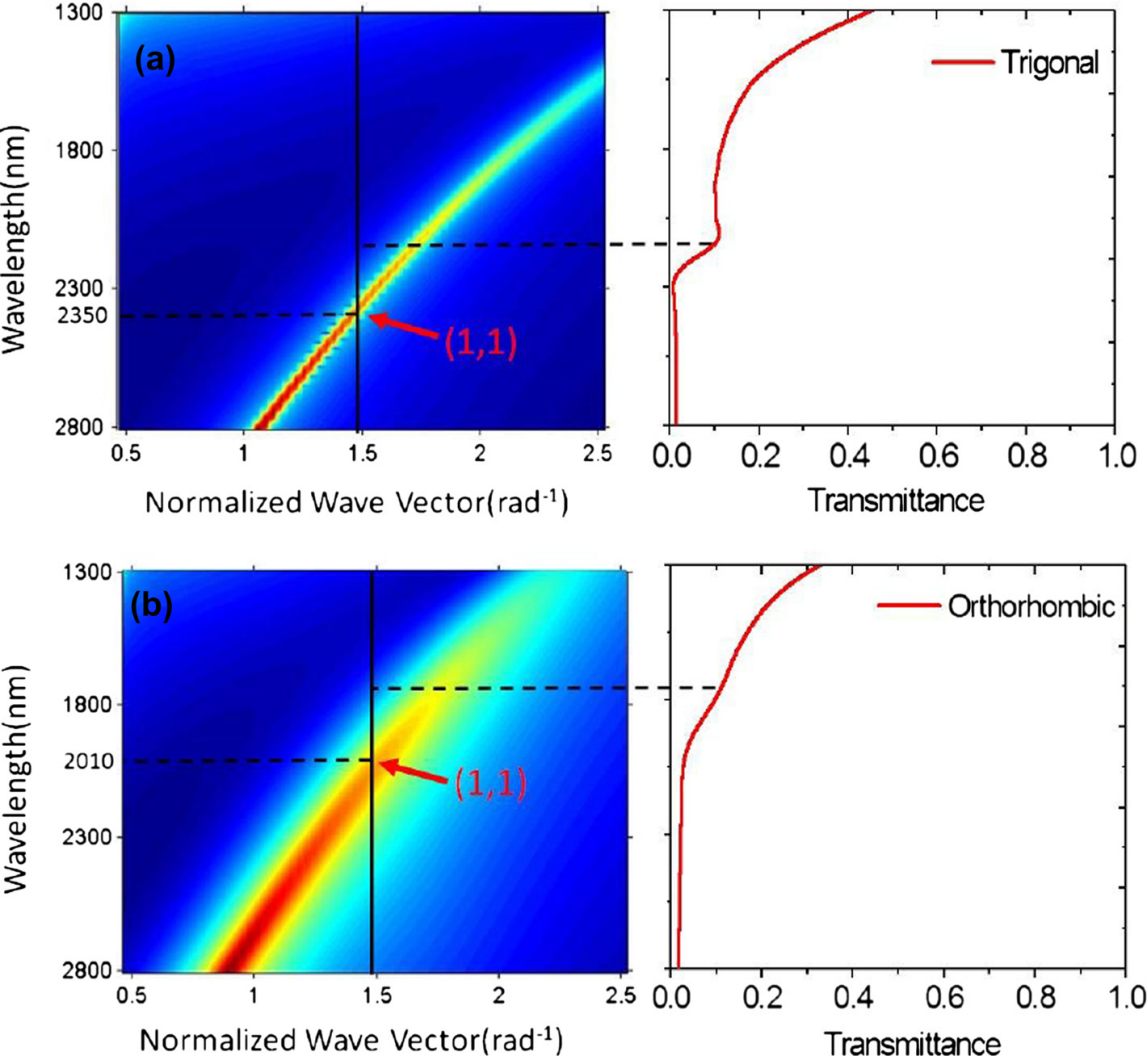
**Dispersion relation of the structure.** Representation of the dispersion relation of the Au-Bi_2_Se_3_-Au trilayer (left) and the transmittance of the multilayer metamaterials (right) for both **(a)** trigonal Bi_2_Se_3_ and **(b)** orthorhombic Bi_2_Se_3_.

To further understand the negative-index resonance in the metamaterials, it is useful to study the dispersion of the surface plasmon polariton (SPP) modes within the multilayer structure. Both the internal and external SPP modes in the multilayer metamaterials are similar to those of the same structure without resonant elements, i.e., MDM films [42], where the internal SPP mode resonates in the inner surfaces of the metal layers and the external SPP mode resonates in the outer surfaces of the metal layers. Therefore, the SPP dispersion relation of the multilayer metamaterial can be approximately approached by that of the MDM structure. In Figure 8, we have calculated the SPP mode dispersion relation of the Au-Bi_2_Se_3_-Au sheets with the top Au film thickness *t*_1_ = 30 nm, middle Bi_2_Se_3_ film thickness *t*_2_ = 60 nm, and bottom Au film thickness *t*_3_ = 30 nm. The transmittance spectrum of the multilayer metamaterials is also depicted together with the dispersion relation of the Au-Bi_2_Se_3_-Au films.

Recalling the coupling condition from light to SPP modes [42], it can be seen that the (1,1) internal resonance of the Au-Bi_2_Se_3_-Au trilayer is excited at 2,350 nm associated with the trigonal Bi_2_Se_3_ in Figure 8a. This internal SPP resonance blueshifts to 2,010 nm when the trigonal state changes to the orthorhombic state as shown in Figure 8b. We also observe that the two internal (1,1) modes which appear at 2,350 and 2,010 nm in the simple MDM structure do not perfectly match the two absorbance peaks at the resonance wavelengths of 2,140 and 1,770 nm in the multilayer metamaterials for both the trigonal and orthorhombic phases, respectively. This difference is because the dispersion relation of the SPP modes used as matching condition does not include the resonant squares, which cause a resonance shift [42].

## Conclusions

In conclusion, this work numerically demonstrates the tunable optical properties of an ENA perforated through Au/Bi_2_Se_3_/Au trilayers. We present that the MDM-ENA can be improved to exhibit a substantial frequency tunability of the intrinsic resonance in the NIR spectral region by selecting Bi_2_Se_3_ as the active dielectric material. Particularly, the resonant transmission, reflection, and the effective constitutive parameters of the Bi_2_Se_3_-coupled multilayer MM can be massively blueshifted by transiting the phase of the Bi_2_Se_3_ film from the trigonal to orthorhombic. This may offer an innovative and practical paradigm for the development of tunable photonic devices. We expect that our results will facilitate further experimental studies of the tunable MMs and make this technique suitable for tuning of plasmon resonance in the optical regime.

## Competing interests

The authors declare that they have no competing interests.

## Authors' contributions

TC conceived the idea of using topological insulator for tuning the resonance in the metamaterials, designed the metamaterial, and wrote the manuscript. SW carried out the simulations and prepared the figures. Both authors read and approved the final manuscript.
